# Left atrial appendage occlusion versus NOACs in atrial fibrillation: Which way is the weight of evidence tilting in 2026?

**DOI:** 10.1016/j.ipej.2026.03.007

**Published:** 2026-03-03

**Authors:** Sivaram Neppala, Adil Ahmed, Priyansh Patel, Hemal M. Nayak

**Affiliations:** a The University of Texas at San Antonio, San Antonio, TX, USA; b University of Miami Miller School of Medicine, Miami, FL, USA

## Abstract

Preventing stroke is a key aspect of managing atrial fibrillation (AF). Non–vitamin K antagonist oral anticoagulants (NOACs) have become the preferred choice due to their improved safety profiles and ease of use compared to warfarin. At the same time, left atrial appendage occlusion (LAAO) has evolved from being an option only for patients unable to tolerate anticoagulation to a legitimate treatment supported by clinical trials, longer-term follow-up, and real-world data. As ongoing research progresses, clinicians are increasingly considering whether LAAO could potentially replace long-term anticoagulation. This review analyzes comparative evidence on NOACs versus LAAO through 2026, focusing on stroke prevention, bleeding complications, survival, procedural risks, long-term outcomes, and appropriate patient selection. Special attention is given to the Indian subcontinent, where bleeding tendencies, coexisting conditions, medication adherence, and access to medical care create unique challenges compared to Western populations. Available evidence shows NOACs remain the standard initial therapy for most AF patients. However, LAAO delivers an important alternative for selected individuals at elevated bleeding risk, those experiencing recurrent clots despite proper anticoagulation, or patients unable to maintain lifelong drug treatment.

## 1 Introduction

Atrial fibrillation represents the most frequently observed sustained cardiac arrhythmia in clinical medicine and remains a leading cause of ischemic stroke globally. Population-based investigations have repeatedly demonstrated that AF increases stroke risk approximately fivefold, even after accounting for other vascular risk factors [1]. Strokes occurring in the setting of AF demonstrate greater severity, produce more substantial disability, and carry higher mortality than strokes arising from alternative mechanisms (see Table 1).

**Table 1:** LAAO versus NOACs: comparative domains influencing therapy selection in 2026.

Domain	NOAC therapy	LAAO
Stroke/systemic embolism prevention	Strong evidence base; first line for most patients	Comparable ischemic stroke prevention in selected patients; strongest RCT data from PRAGUE-17
Hemorrhagic stroke	Reduced vs warfarin, but bleeding risk persists	Typically, lower hemorrhagic stroke after completion of post-implant therapy, consistent across long-term trial follow-up
Major bleeding	Lower intracranial hemorrhage than warfarin, but GI bleeding and frailty-related bleeding remain concerns	Long-term reduction in major/nonprocedural bleeding is a principal advantage; early bleeding risk depends on post-implant regimen
Upfront risk	No procedure: bleeding risk begins immediately with therapy	Procedural risk (pericardial effusion, vascular complications, peri-procedural stroke) but low in contemporary practice
Long-term burden	Lifelong adherence required; missed doses rapidly reduce protection	Typically limited antithrombotic course; requires surveillance imaging and early follow-up (and management of DRT/leak if present)
Renal dysfunction	Dosing/agent selection limited; both thrombotic and bleeding risk may rise	Avoids indefinite systemic anticoagulation but still requires short-term post-implant therapy; careful selection needed
Adherence feasibility	Dependent on patient adherence; affected by cost and access	Less dependent on daily adherence after endothelialization, but depends on procedural access and follow-up imaging
Guideline positioning	First-line for most eligible patients	Guidelines support LAAO for contraindication to long-term OAC (Class 2a) and as an alternative in high bleeding risk (Class 2b)

In AF, thromboembolic events arise predominantly from blood stasis within the left atrial appendage. Evidence from autopsy series, transesophageal echocardiographic investigations, and intraoperative observations indicates that over 90% of intracardiac thrombi in AF patients form within the left atrial appendage [2]. This anatomical predilection provides the mechanistic foundation for two distinct preventive approaches: systemic anticoagulation and mechanical appendage exclusion.

Treatment options for AF have undergone substantial transformation. Vitamin K antagonists had long served as the sole effective oral anticoagulant class, yet their clinical use was constrained by narrow therapeutic indices, mandatory serial monitoring, numerous dietary and pharmacologic interactions, and unpredictable anticoagulant responses. The development of NOACs represented a major therapeutic advance. Pivotal randomized controlled trials demonstrated comparable or superior efficacy and safety, leading to broad clinical adoption and incorporation into practice guidelines [[3], [4], [5], [6], [7]].

Concurrently, percutaneous LAAO devices emerged as a mechanical or procedural alternative to exclude the left atrial appendage from the systemic circulation. Originally indicated for patients with contraindications to long-term anticoagulation, LAAO has progressively strengthened its evidence base for both efficacy and safety. Current clinical practice requires careful consideration of multiple effective NOAC agents alongside an expanding body of evidence supporting LAAO as a viable therapeutic strategy.

### 1.1 Pathological basis, mechanistic differences: systemic anticoagulation versus mechanical exclusion

**Fig. 1:**
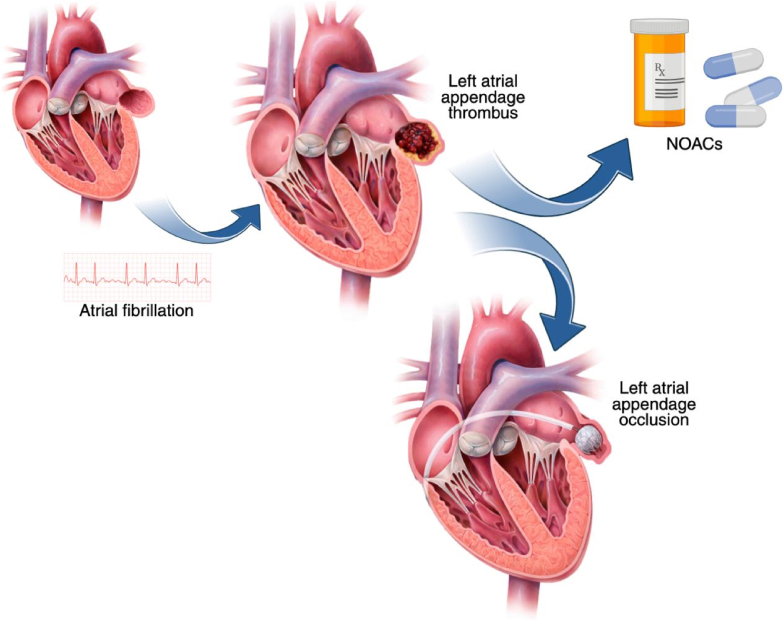
Pathophysiologic rationale for stroke prevention in atrial fibrillation. Schematic shows the basis for prevention and differences between systemic anticoagulation with NOACs and mechanical LAAO.

Non–vitamin K antagonist oral anticoagulants (NOACs) and left atrial appendage occlusion (LAAO) both reduce thromboembolic risk in atrial fibrillation through fundamentally different mechanisms, resulting in distinct clinical trade-offs (Figure-1).

### 1.2 Systemic anticoagulation

**Fig. 2:**
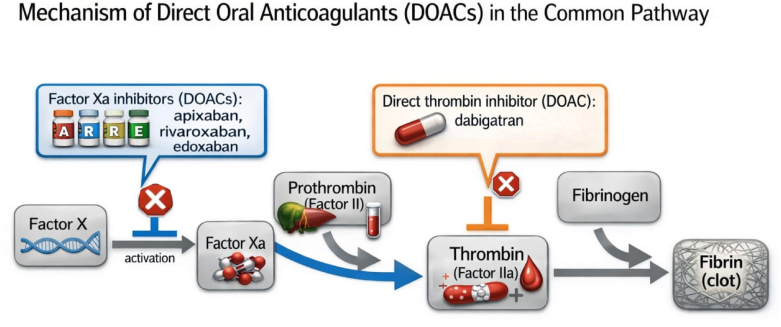
Mechanism of action of NOACs within the coagulation cascade, highlighting factor Xa and direct thrombin inhibition.

NOACs exert their effects through systemic inhibition of specific coagulation factors, either thrombin (factor IIa) or factor Xa, thereby reducing thrombus formation. (Figure-2). While this systemic anticoagulant effect provides protection against stroke, it also predisposes patients to bleeding complications. Although NOACs demonstrate a substantially lower risk of intracranial hemorrhage compared with warfarin, gastrointestinal bleeding complications can still occur, particularly among certain populations, such as the elderly, and patients with chronic kidney disease and anemia [8,9].

### 1.3 Mechanical exclusion

In contrast, LAAO directly targets the anatomical source of thrombus formation in atrial fibrillation by mechanically excluding the left atrial appendage from systemic circulation. Following device implantation and endothelialization, long-term anticoagulation is often no longer needed. This approach shifts the risk from chronic bleeding toward procedural complications. Early LAAO procedures were associated with significant periprocedural risks, such as pericardial effusion and device embolization. However, contemporary outcomes have improved due to advances in design, refined implantation techniques, standardized imaging protocols, and operator experience, resulting in far fewer complications [10].

## 2 Evidence supporting NOAC therapy

### 2.1 Randomized trial evidence

Four key randomized controlled trials (RCTs) compared dabigatran, rivaroxaban, apixaban, and edoxaban collectively to warfarin in more than 70,000 patients with atrial fibrillation. Each trial demonstrated noninferiority or superior efficacy compared with warfarin for the prevention of stroke and systemic embolism [[4], [5], [6], [7]]. Meta-analyses of these trials revealed that NOACs were associated with significantly lower rates of intracranial bleeding and all-cause mortality compared to warfarin, although a modest increase in gastrointestinal bleeding was observed [3].

### 2.2 Observational evidence

Large observational registries have corroborated the RCT findings across diverse populations, including elderly patients and those with prior cerebrovascular events. However, real-world data highlight issues that may have been overlooked in such trials, such as suboptimal adherence or premature therapy discontinuation [[11], [12], [13]]. These challenges are further amplified in fragmented healthcare systems with resource limitations.

### 2.3 Limitations of NOAC therapy

Despite their advantages, several factors limit the effectiveness of long-term NOAC therapy. Bleeding remains the principal limitation, with risk increasing in patients with history of prior bleeding, gastrointestinal disease, or frailty [9,14]. Renal dysfunction, particularly common in the elderly population, complicates dosing and increases both bleeding and thrombotic risk [8]. Adherence is also very important, given the short half-lives of NOACs. Missed doses or under-dosing can rapidly diminish therapeutic effectiveness. Additionally, financial barriers further restrict access in many low- and middle-income countries. Finally, ischemic strokes occasionally occur despite therapeutic anticoagulation.

## 3 Evolution of left atrial appendage occlusion

### 3.1 Early trials

PROTECT AF was the first randomized trial demonstrating noninferiority of percutaneous LAAO compared with warfarin for the composite endpoint of stroke, systemic embolism, and cardiovascular death [15]. However, significant procedural complications limited its early adoption. The PREVAIL trial aimed to replicate these results with improved procedural safety [16]. The trial achieved its efficacy endpoints with fewer complications. Long-term follow-up data from both studies revealed significant benefits, including a reduction in hemorrhagic stroke and cardiovascular mortality compared to warfarin [17,18].

### 3.2 LAAO compared with NOACs

The PRAGUE-17 trial directly compared LAAO with NOAC therapy in high-risk patients with atrial fibrillation [19]. LAAO performed comparably to NOACs for the composite outcome of stroke, systemic embolism, major bleeding, and cardiovascular death. An extended follow-up reinforced these findings and suggested fewer bleeding events in the LAAO group [20].

### 3.3 Registry data

Large contemporary registries, including EWOLUTION and the National Cardiovascular Data Registry (NCDR) LAAO Registry, reflect current practice and demonstrate implantation success rates exceeding 95%, accompanied by significant reductions in major procedural complications [[21], [22], [23]].

## 4 Comparative outcomes: LAAO versus NOACs

### 4.1 Stroke and systemic embolism

**Fig. 3:**
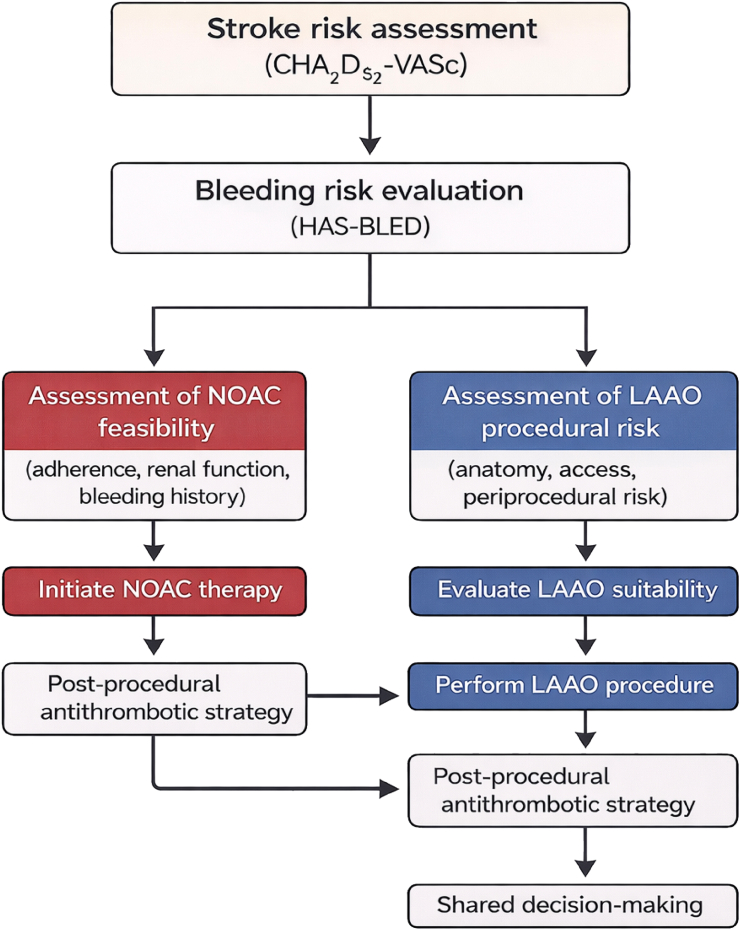
Clinical decision framework for stroke prevention in atrial fibrillation.

Randomized trials and registry analyses demonstrate comparable rates of ischemic stroke between LAAO and NOAC therapy in appropriately selected patients (Fig. 3). Differences emerge when stroke subtypes are compared, with consistently lower rates of hemorrhagic stroke following LAAO [17,24].

### 4.2 Bleeding outcomes

The most significant advantage of LAAO appears in bleeding outcomes. Long-term data indicate reduced rates of major and fatal bleeding, particularly among patients with a history of hemorrhage or elevated bleeding risk [24,25].

### 4.3 Mortality

Although individual trials were underpowered to detect differences in mortality, pooled analyses suggest that LAAO reduces cardiovascular and all-cause mortality over extended follow-up, largely attributable to fewer fatal bleeding events [17,18,26].

## 5 Evidence synthesis from contemporary meta-analyses

Although individual randomized control trials provide the highest level of evidence, we should use pooled analyses that integrate early warfarin-comparator trials with contemporary trials on NOACs. PRAGUE-17 remains the principal head-to-head RCT of percutaneous LAAO vs. NOAC therapy, demonstrating noninferiority for a composite of major cardiovascular, neurologic, and bleeding events with sustained noninferiority at long-term follow-up and a signal towards fewer nonprocedural bleeding events over time [19,20].

**Fig. 4:**
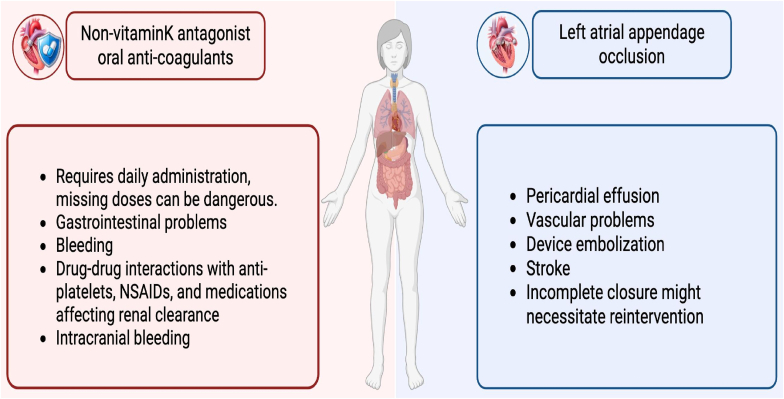
Comparative risks associated with Non-vitamin K oral anticoagulants versus Left atrial appendage occlusion.

In parallel, meta-analyses that pool randomized data comparing LAAO with oral anticoagulation (including NOACs) support three key observations: (1) ischemic stroke/systemic embolism rates are broadly similar between the two strategies in appropriately selected patients [17,24]; (2) hemorrhagic stroke and long-term bleeding tend to favor LAAO given the reduced lifetime exposure to systemic anticoagulation once post-thrombotic therapy is completed [24,25]; and (3) mortality differences appear largely driven by reductions in fatal bleeding and hemorrhagic stroke rather than by differences in ischemic stroke alone [17,18,26]. Another commonly cited meta-analysis has shown comparable stroke prevention with LAAO versus anticoagulation while highlighting reductions in hemorrhagic stroke and late bleeding outcomes in patients with LAAO devices (Fig. 4). These syntheses reinforce a central theme: the clinical advantage of using LAAO is less about superior ischemic stroke prevention and more about shifting the long-term risk profile away from major bleeding in patients with elevated risk.

## 6 Patient selection: matching therapy to risk profile

### 6.1 Candidates for NOAC therapy

• Low to moderate bleeding risk
• Reliable medication adherence
• Stable renal function
• No major bleeding history
• Patient preference for medication

### 6.2 Candidates for LAAO

• Prior intracranial hemorrhage
• Recurrent major bleeding despite management optimization
• Thromboembolism while on therapeutic anticoagulation
• Contraindications to long-term anticoagulation
• High occupational bleeding risk
• Patient preference after informed discussion

**Fig. 5:**
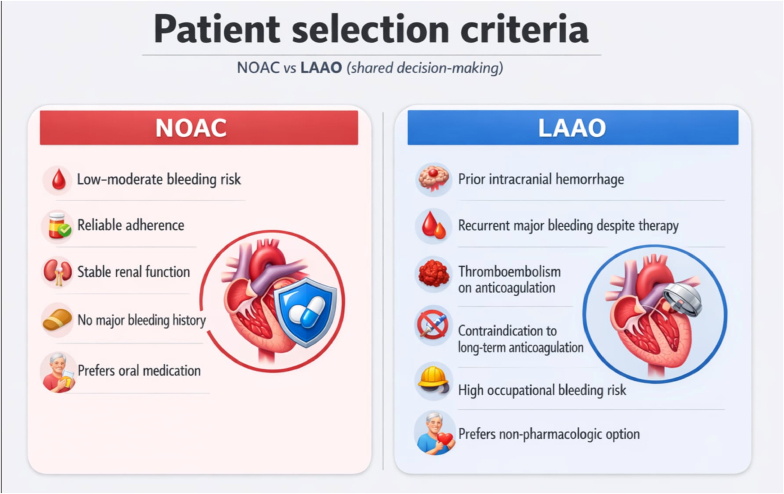
Clinical characteristics of systemic anticoagulation with non-vitamin K oral anticoagulants (NOACs) versus left atrial appendage occlusion (LAAO) for stroke prevention in patients with atrial fibrillation.

Contemporary consensus statements emphasize individualized decision-making guided by clinical risk profiles. [27,28] (Fig. 5).

## 7 Context of the Indian subcontinent

The patient population in India differs significantly from that in Western cohorts in several key respects. They tend to be younger at presentation and exhibit higher prevalence of structural heart disease, anemia, and renal dysfunction [29,30]. Out-of-pocket healthcare expenses significantly impact adherence to lifelong NOAC therapy. While access to LAAO is expanding, it remains largely limited to tertiary centers.

For individuals in whom sustained anticoagulation is unsafe or impractical, LAAO may represent a particularly valuable alternative. Regional considerations—including affordability, access to follow-up care, and patient education—must guide therapeutic decisions.

## 8 Framework for clinical decision-making

Clinicians should discuss the following elements with patients when deciding between NOAC therapy and LAAO:

1. Stroke and Bleeding Risk Assessment: Calculate the patient's CHA_2_DS_2_-VASc score to assess stroke risk and utilize HAS-BLED or other validated tools to evaluate bleeding risk. Review the patient's history for bleeding events, falls, or risk factors such as age, kidney or liver disease, antiplatelet therapy, and alcohol consumption (Fig. 3).
2. NOAC Therapy: Benefits and Limitations: NOACs reduce stroke risk by 60-70%, matching or surpassing warfarin, but they need daily administration. Missing doses and under-dosing can be dangerous. Side effects include gastrointestinal problems and bleeding. Be cautious of drug-drug interactions, especially with antiplatelets, NSAIDs, and medications affecting renal clearance. Although intracranial bleeding is less common than with warfarin, gastrointestinal bleeding occurs more frequently. While strokes can still occur, proper anticoagulation significantly lowers the likelihood (Fig. 4).
3. LAAO: Procedural Risks and Long-Term Outcomes: Describe the transseptal puncture, device deployment with fluoroscopy and echocardiography, and the typical hospital stay. Mention risks such as pericardial effusion (1-2%), vascular issues, rare device embolization, and the approximate 0.5% risk of stroke. Patients should be aware that 1-3 months of anticoagulation or 6 months of dual antiplatelet therapy will still be required post-operatively, but can subsequently be discontinued in favor of aspirin monotherapy. The bleeding risk decreases after a year, and hemorrhagic stroke rates decline with cessation. Rarely, device thrombus may develop, and incomplete closure might necessitate reintervention or continuing anticoagulation (Fig. 4).
4. Post-Procedural Antithrombotic Management: Typically, patients are treated with warfarin or NOAC for 45 days, then switched to dual antiplatelet therapy for six months, followed by aspirin monotherapy indefinitely. Some centers may shorten this duration based on imaging evidence of device endothelialization. Patients intolerant to antiplatelet therapy may still be suitable candidates, although data is limited. Follow-up imaging is advised at 45 days and 6 to 12 months to verify proper device placement and closure. There are data that support omitting the 45 days of warfarin or NOAC therapy and utilizing DAPT immediately after LAAO implantation.
5. Cost, Access, and Practical Considerations: Compare the lifelong costs of NOAC therapy versus a one-time LAAO. NOACs cost ₹3000- ₹ 8000 per month (₹36,000- ₹ 96,000 annually), whereas LAAO costs ₹3- ₹ 5 lakh as a single expense. Coverage varies. NOACs are accessible everywhere; LAAO requires travel to specialized centers. NOACs need regular follow-up, while LAAO requires fewer visits. Consider adherence, access, and affordability.
6. Patient Preferences and Values: Ask the patient how comfortable they feel with medication compared to a procedure and inquire about any concerns about potential risks. Consider their job, daily habits, and the level of risk they're willing to accept. Honour their preferences when both options are appropriate.

Adjust discussions to consider patient factors such as age, health status, and access. For example, a 55-year-old with previous gastrointestinal bleeding might opt for LAAO, whereas an 80-year-old with multiple health issues may prefer NOACs. Patients in rural areas may also lean toward NOACs despite concerns about bleeding. These practical factors, along with patient preferences and values, should guide shared decision-making.

## 9 Device evolution and procedural standardization

The safety and performance profile of LAAO devices reflects not only increased operator experience but also significant advances in device engineering and procedural standardization. Earlier devices were associated with a higher learning curve for successful deployment and higher rates of periprocedural complications. Newer devices, such as the Watchman FLX, incorporate design features intended to improve conformability, anchoring, and sealing, thereby reducing the risk of peri-device leak, embolization, and device-related thrombi. Real-world data suggest that procedural success rates can exceed 95% among experienced operators, with significantly lower rates of post-procedural complications than with early device implants [21,22].

Additionally, registry datasets suggest that outcomes are strongly volume-dependent and improve with standardized imaging workflows and operator experience [21,22]. Recent large-scale registry analyses of contemporary devices, such as the Watchman, demonstrate low early rates of ischemic stroke and periprocedural complications, including pericardial effusion, supporting the conclusion that LAAO techniques have matured into a predictable structural intervention for appropriately selected patients [23]. Comparative analyses of newer-generation versus first-generation devices also support improved periprocedural safety consistent with the theme that the “procedural penalty” of LAAO has substantially decreased over time [12].

## 10 Device platforms and comparative clinical outcomes

Two FDA-approved device platforms currently lead the field of percutaneous left atrial appendage occlusion: WATCHMAN (including WATCHMAN FLX) and Amplatzer Amulet. The devices employ distinct structural approaches: WATCHMAN uses a plug-based architecture, whereas Amulet features a dual-seal “pacifier” configuration. The Amulet IDE randomized trial demonstrated that Amulet was noninferior in safety and effectiveness compared with WATCHMAN 2.5, with superior complete occlusion rates. However, the trial revealed differing profiles in periprocedural complications, bleeding events, and peridevice leak characteristics [31]. These performance differences inform clinical device selection, which depends on patient-specific anatomical features and risk factors. Key considerations include ostial dimensions, landing zone characteristics, lobar anatomy complexity, and institutional expertise with each device's deployment technique [31].

The SWISS-APERO randomized trial offered a direct, imaging-based comparison between Amulet and WATCHMAN (primarily WATCHMAN FLX). At 45 days, Amulet did not demonstrate a reduction in the composite endpoint of device crossover or residual left atrial appendage patency on cardiac CT compared with WATCHMAN. However, Amulet showed fewer peri-device leaks on transesophageal echocardiography (none exceeding 5 mm), although this was accompanied by increased major procedure-related complications, predominantly bleeding. Short-term clinical outcomes remained comparable between the two devices [32]. Extended follow-up data from SWISS-APERO, published in *JACC* in 2025, revealed no statistically significant difference in the prespecified 3-year composite endpoint of cardiovascular death, stroke, transient ischemic attack, or systemic embolism. The point estimate favored Amulet numerically, and this difference was statistically significant in both the as-treated and per-protocol analyses, which investigators characterized as hypothesis-generating findings [33]. Registry data from the NCDR LAAO Registry provides additional context for WATCHMAN FLX performance in routine clinical practice. These real-world data demonstrate high procedural success rates and low complication rates, confirming the device's feasibility and safety profile when deployed across diverse practice settings [34].

Future developments in device technology and procedural techniques address three persistent challenges: peridevice leakage, device-related thrombus formation, and deployment and recapture difficulties. Newer devices incorporate improved conformability and sealing mechanisms, while procedural advances focus on enhanced visualization and coaxial deployment techniques. Material science innovations aim to achieve more predictable healing responses and endothelialization. A recent comprehensive review outlines these advances across device engineering, procedural refinement through intracardiac echocardiography and CT-guided workflows, and computational modeling combined with advanced imaging to minimize malposition-related complications [35].

The clinical application of left atrial appendage occlusion continues to broaden beyond traditional indications. Randomized trial evidence now supports combining LAAO with rhythm-control strategies. In patients undergoing atrial fibrillation ablation, LAAO reduced non-procedural bleeding events compared with continued oral anticoagulation while maintaining comparable outcomes for major ischemic events during follow-up [36].

## 11 Post-procedural antithrombotic management

Post-implant antithrombotic therapy remains a key determinant of early outcomes after LAAO and a major reason most guidelines remain conservative in broadly advocating for LAAO as a complete replacement for systemic anticoagulation. Traditional protocols evolved from early Watchman trials and commonly included short-term anticoagulation, followed by dual antiplatelet therapy, and then indefinite single antiplatelet therapy. However, contemporary practice should be individualized, particularly for patients who were initially referred for LAAO because of their elevated bleeding risk.

Expert consensus statements acknowledge the individual variability in post-procedural antithrombotic regimens and recommend personalized tailoring based on bleeding risk, device type, and follow-up imaging findings [27]. Data from national registries suggest that DAPT-based approaches are effective in selected patients undergoing LAAO, aiming to reduce the risk of early post-procedural bleeding in those at elevated risk while maintaining acceptable thrombotic outcomes [13]. Early adverse event rates for ischemic stroke or pericardial effusion remain low, but early major bleeding is still clinically relevant, emphasizing that standardized post-implant antithrombotic therapy may not be ideal in all cases. Further studies are necessary to refine the optimal post-implant antithrombotic therapy in carefully selected patients, with emphasis on image-guided discontinuation of therapy. Until then, management should be framed as a core component of shared-decision making rather than a procedural afterthought.

## 12 Future directions

Ongoing trials such as OPTION, CHAMPION-AF, and CATALYST are evaluating LAAO in broader patient populations, including those without formal contraindications to anticoagulation. Additionally, optimal post-implant antithrombotic regimens remain an area of active investigation, with emerging interest in personalized, image-guided strategies [[37], [38], [39]].

The OPTION and CHAMPION-AF studies compare LAAO with NOACs in patients without contraindications [37,38]. They aim to determine whether LAAO can serve as a first-line treatment or is only suitable for patients who cannot tolerate anticoagulation, particularly those with elevated bleeding risk. Post-procedure antithrombotic treatment protocols still require development. The CATALYST trial investigates whether antiplatelet therapy alone is sufficient for patients unable to take anticoagulants [39]. Current guidelines—45 days of anticoagulation, followed by six months of dual antiplatelet therapy, then aspirin—are based on initial research rather than systematic validation. Personalized, imaging-guided approaches might better balance the need to prevent thrombus formation with the risk of bleeding [40]. There is limited data on cost-effectiveness in low- and middle-income countries; models developed in high-income countries do not apply directly to India, where NOACs impose a significant financial burden on families and LAAO requires travel to specialized centers. Regional analyses could help optimize resource use. Access barriers continue to limit both therapies: NOACs remain unaffordable for many patients, and LAAO is available only in major cities with tertiary centers. Expanding training programs to smaller urban areas could help reduce health disparities [41]. Establishing an Indian LAAO Registry would facilitate local tracking of outcomes and complications, providing more relevant data than its Western counterparts. High-risk groups, such as those over the age of 80, patients with severe renal impairment, stroke survivors, or those with multiple comorbidities, are critically underrepresented in clinical trials. Incorporating patient-reported outcomes alongside traditional measures may enhance understanding. Future investigations will clarify whether LAAO will evolve into a standard therapy for wider patient populations or continue to serve a more selective role.

## 13 Conclusion

NOACs continue to remain the foundation of preventing strokes in patients with atrial fibrillation. Nevertheless, emerging evidence indicates an expanding role for LAAO in carefully selected patients. Current evidence does not support universal replacement of NOAC therapy but increasingly favors earlier consideration of LAAO in individuals at elevated bleeding risk, those with thromboembolic events despite anticoagulation, or patients unable to adhere to lifelong drug therapy. For clinicians practicing in India, identifying appropriate candidates and determining optimal timing for LAAO will be central to translating evolving evidence into meaningful improvements in patient outcomes.

## Patient consent

Our manuscript is a review article and does not require patient consent. No identifiable patient information was utilized in writing this review.

## Ethical statement

Our manuscript is a review article and does not require an ethical statement.

## Author statement

Co-Authors Sivaram Neppala, MD and Adil Ahmed, MD are co-first authors and were responsible for conducting a literature review, writing the review article and conceptualizing the figures. Co-Author Priyansh Patel was responsible for creating the figures in the manuscript, Co-Author Hemal M. Nayak, MD, MBA was responsible for the entire manuscript including concept, writing, editorial review, conceptualization and editing of the figures.

Sivaram Neppala, MD.

The University of Texas at San Antonio, San Antonio, TX, USA.

Adil Ahmed, MD.

The University of Texas at San Antonio, San Antonio, TX, USA.

Priyansh Patel.

University of Miami Miller School of Medicine, Miami, Florida, USA.

Hemal M. Nayak, MD, MBA.

The University of Texas at San Antonio, San Antonio, TX, USA.

## Declaration of competing interest

The authors declare that they have no known competing financial interests or personal relationships that could have appeared to influence the work reported in this paper.
